# Radiographic evaluation of congruency of the first metatarsophalangeal joint in hallux valgus

**DOI:** 10.1186/s13018-022-03028-1

**Published:** 2022-03-05

**Authors:** Yan Li, Xu Tao, Kanglai Tang

**Affiliations:** Department of Orthopedics/Sports Medicine Center, The First Affiliated Hospital of Army Medical University (Southwest Hospital of Gaotanyan Zhengjie), Shapingba District, Chongqing, 400038 China

**Keywords:** Hallux valgus; Metatarsophalangeal joint; Congruency; Metatarsophalangeal joint angle; Congruency index

## Abstract

**Background:**

Congruency of the first metatarsophalangeal (MTP) joint is extremely important for the selection of surgical methods and prognosis, while radiographic evaluation methods are relatively lacking. The purpose of this article was to explore radiographic indicators for evaluating congruency of the first MTP joint.

**Methods:**

We selected patients with hallux valgus who had a weightbearing X-ray in the outpatient system and measured their hallux valgus angle (HVA). In total, 183 cases of 245 feet with HVA greater than 15° were selected. The distal metatarsal articular angle (DMAA), metatarsophalangeal joint angle (MTPJA), congruency index (CI) and tibial sesamoid position (TSP) were measured and statistically analysed.

**Results:**

The higher the degree of hallux valgus was, the higher the proportion of incongruency of the first MTP joint. Significant differences were found in the DMAA, MTPJA and CI between the congruency and incongruency groups of patients with moderate-to-severe hallux valgus (*P* < 0.05). The areas under the curve (AUCs) of the receiver operating characteristic (ROC) curve for DMAA was 0.554 (*P* > 0.05). However, the MTPJA and CI were 0.906 and 0.884, the sensitivity values reached 0.791 and 0.949, the specificity values were 0.862 and 0.644, and the critical values were 10.67 and 0.765, respectively. The correlation test indicated that in the congruency group, the DMAA and HVA were positively correlated, but the MTPJA, CI and HVA had low correlation coefficients. The DMAA and HVA were not correlated in the incongruency group; however, the MTPJA and HVA were significantly positively correlated, and the CI and HVA showed a negative correlation (*P* < 0.05).

**Conclusion:**

The MTPJA and CI are indicators that can be used to quantitatively evaluate the congruency of the first MTP joint, taking 10° and 0.765 as the demarcation points, respectively. Clinically, congruency of the MTP joint should be considered when choosing surgical methods for different degrees of hallux valgus, and the MTPJA and CI can be used as quantitative evaluation indicators.

Level of evidence: Level III, Retrospective Comparative Study.

## Background

Congruency of the first metatarsophalangeal (MTP) joint is important for the choice of hallux valgus surgery and post-operative recurrence [1]. However, the data on the congruency of the MTP joint are quite different [2, 3]. Incongruency of the MTP joint affects a higher proportion of patients with moderate-to-severe hallux valgus. For patients with hallux valgus of the same degree, there are large differences in the choice of surgical methods due to the existence of MTP joint congruency and incongruency. For example, double metatarsal osteotomy (DMO) is often required for patients who have a large hallux valgus angle (HVA) and congruency of the MTP joint [4, 5]. For patients with moderate-to-severe hallux valgus with incongruency metatarsophalangeal joints, scarf osteotomy or simple Chevron osteotomy is effective [6, 7].The previous literature used mostly the congruency or incongruency metatarsophalangeal joint as an index to evaluate the effect of hallux valgus surgery [8–10], and its measurement rests only on the arc ratio of the articular surface; however, there is no quantitative measurement index for the congruency of the first MTP joint [11].

The purpose of this article was to quantitatively evaluate the congruency of the MTP joint through two innovative indicators and conduct diagnostic experimental analysis to explore the imaging indicators of MTP joint, aiming to assess the evaluation value of the two new indicators and the intrinsic relationship between different MTP joint imaging indicators. The results will provide a basis for surgical selection of patients with different MTP joints and add quantitative indicators to evaluate MTP joints for post-operative follow-up.

## Methods

Patients who had a weightbearing X-ray in the outpatient system from January 2018 to January 2021 were selected. HVA was measured in a PACS system. The inclusion criteria were age ≥ 18 years; patients who underwent foot-bearing X-ray examination in the outpatient system; and patients who measured HVA > 15°. The exclusion criteria were patients aged < 18 years; patients with a history of foot and ankle trauma or fracture; or patients with a history of previous foot or ankle surgery. Finally, 183 hallux valgus patients with 245 feet were included, comprising 51 males and 132 females. Among the patients, 131 feet (53.47%) had mild hallux valgus (HVA: 15–30°), 80 feet (32.65%) had moderate hallux valgus (HVA: 31–40°), and 34 feet (13.88%) had severe hallux valgus (HVA ≥ 40°) according to the classification criteria for hallux valgus [12].

**Fig. 1:**
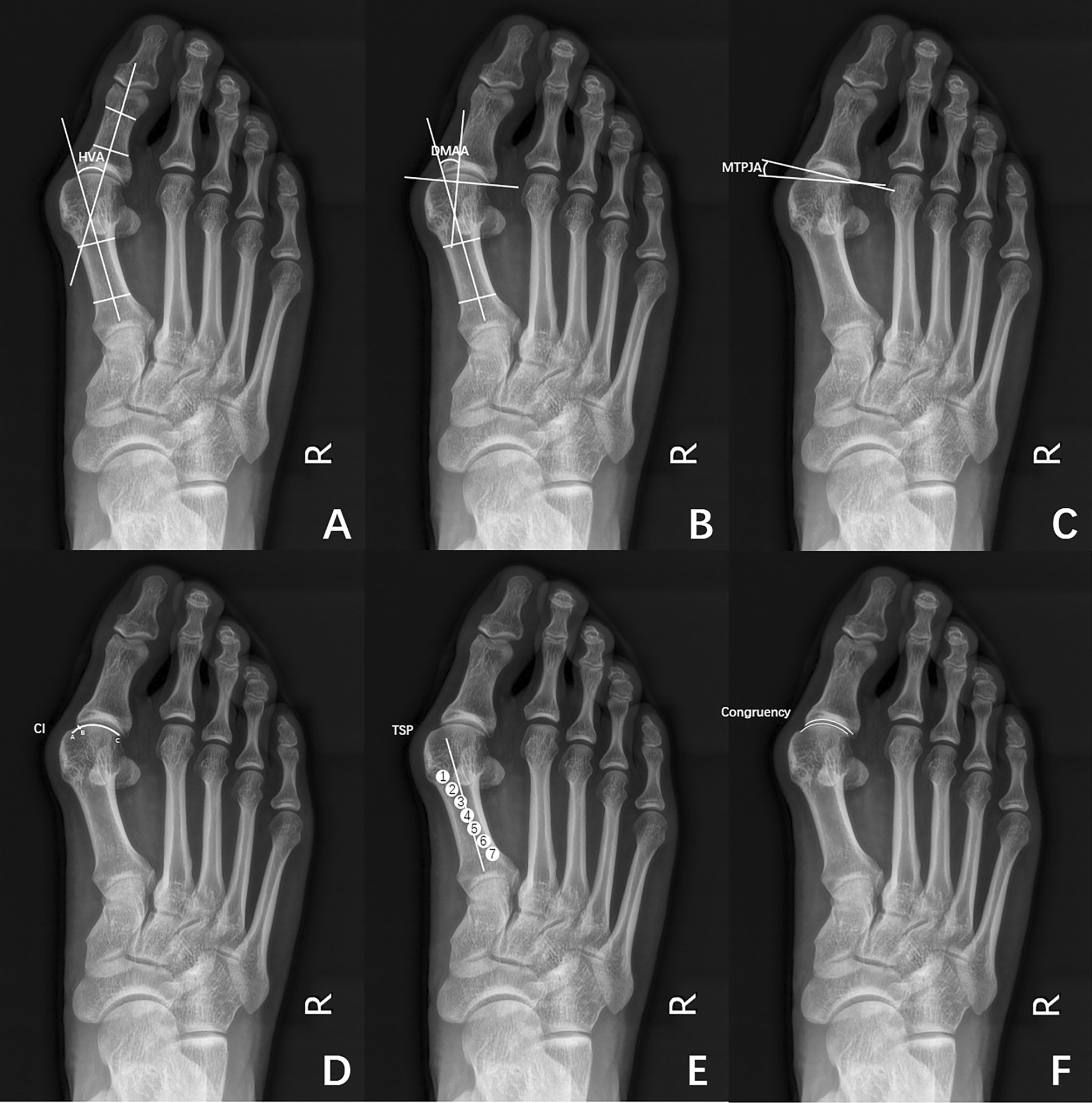
The imaging indicators of the first MTP joint on the weightbearing foot anterior–posterior images. **A** hallux valgus angle, HVA; **B** distal metatarsal articular angle, DMAA; **C** metatarsophalangeal joint angle, MTPJA; **D** congruency index, CI; **E** tibial sesamoid position, TSP; **F** evaluation of the congruency of the first MTP joint

The HVA, distal metatarsal articular angle (DMAA) and tibial sesamoid position (TSP) were measured in weightbearing foot anterior–posterior images according to the measurement method described in previous literature (Fig. 1A, B, E) [13]. According to the congruency and incongruency of the first MTP joint in patients with hallux valgus, we innovatively designed two new measurement indicators, the metatarsophalangeal joint angle (MTPJA) and congruency index (CI), which was defined by the MTPJA as follows: on the weightbearing foot anterior–posterior images, we drew a straight line between the inner and outer edges of the articular surface of the proximal phalanx and the distal metatarsal, respectively, and the angle between the two straight lines was the MTPJA (Fig. 1C). The CI was calculated as described in our previous article [6]. The ratio of the curve length of the metatarsophalangeal joint contact surface to the curve length of the metatarsal head cartilage surface was defined as the CI, which was designed to measure the congruency of the MTP joint (Fig. 1D). There are currently no quantitative indicators to assess whether the metatarsophalangeal joint is congruent; it is based only on the physician's subjective observation of whether the articular surfaces at both ends of the metatarsophalangeal joint were parallel (Fig. 1F). Based on this, we selected two doctors with 20 years of experience in foot and ankle surgery and divided the 245 feet into the congruency and incongruency groups. The HVA, DMAA, MTPJA, CI and TSP were statistically analysed among different degrees of hallux valgus and between the congruency and incongruency groups. Diagnostic tests were performed for the DMAA, MTPJA and CI, and receiver operating characteristic (ROC) curves were plotted. The area under the curve (AUC), sensitivity, specificity and critical value were calculated.

SPSS 20.0 software was used for statistical analysis. Quantitative data were expressed as X ± S. The categorical data were expressed as *n* (%). Comparisons of count data of patients were analysed by means of the Chi-square test. Comparisons of different parameters between patients with different degrees of hallux valgus were analysed by one-way analysis of variance, and the independent sample Student’s *t* test was used to compare different parameters between the congruency and incongruency groups. The diagnostic test of the DMAA, MTPJA and CI used ROC curves and calculated the AUC, critical value, sensitivity, specificity and other parameters. A correlation test was carried out between different parameters of the congruency and incongruency groups, and the correlation coefficient was calculated. The difference was considered to be statistically significant at *P* < 0.05.

## Results

**Fig. 2:**
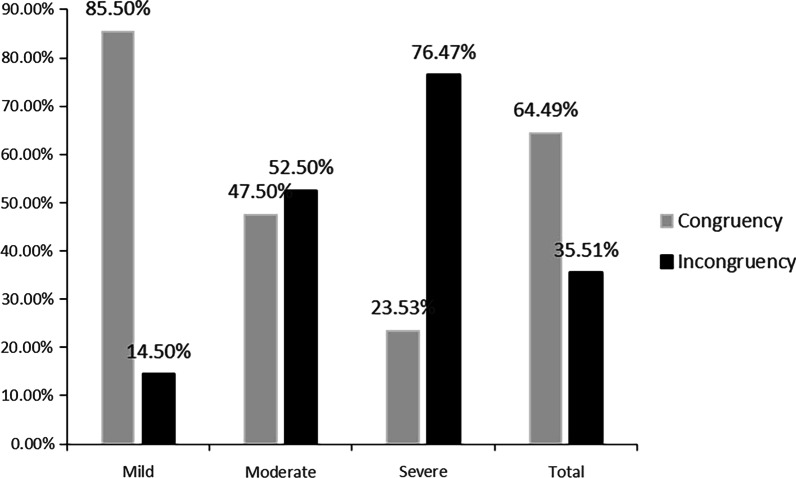
Distribution of congruency of the first metatarsophalangeal joint in patients with mild to severe hallux valgus

The HVA, DMAA, MTPJA and TSP increased significantly as the degree of hallux valgus increased, while the CI decreased (*P* < 0.05) (Table 1). Using the previous observation of the parallelism of the first metatarsophalangeal joint surface as the gold standard [11], we divided 245 feet into the congruency and incongruency groups. In total, approximately 2/3 (64.49%) of the patients had first MTP joints that were congruent, and approximately 1/3 (35.51%) of the patients had first MTP joints that were incongruent. The majority of patients with mild hallux valgus have congruency, while those with severe hallux valgus have incongruency. Patients with moderate hallux valgus were basically the same. The differences among the three groups were statistically significant (*P* < 0.05) (Fig. 2).

**Table 1:** Comparison and analysis of different indexes in patients with different degrees of hallux valgus

Index	Mild hallux valgus	P*	Moderate hallux valgus	P**	Severe hallux valgus	P***
Sex (M/F)	37/70	0.060	15/55	0.637	5/24	0.073
Age (year)	45.60 ± 16.55	0.064	51.44 ± 17.33	0.116	57.34 ± 17.21	0.003
L/R	69/62	0.981	42/38	0.807	17/17	0.781
HVA (°)	24.07 ± 4.01	0.000	33.72 ± 2.79	0.000	46.52 ± 4.71	0.000
DMAA (°)	12.55 ± 5.78	0.000	18.98 ± 7.26	0.040	22.01 ± 10.75	0.000
MTPJA (°)	8.25 ± 4.88	0.000	12.91 ± 7.45	0.000	23.42 ± 13.90	0.000
CI	0.85 ± 0.07	0.000	0.80 ± 0.09	0.000	0.65 ± 0.16	0.000
TSP	3.87 ± 1.65	0.000	5.15 ± 1.67	0.004	6.09 ± 1.08	0.000
Feet number (ratio)	131 (53.47%)	0.003	80 (32.65%)	0.000	34 (13.88%)	0.000
Congruency/incongruency	112/19	0.000	38/42	0.017	8/26	0.000

*HVA* hallux valgus angle, *DMAA* distal metatarsal articular angle, *MTPJA* the first metatarsophalangeal joint angle, *CI* congruency index, *TSP* tibial sesamoid position

P*: comparisons between mild and moderate hallux valgus. P**: comparisons between moderate and severe hallux valgus. P***: comparisons between mild and severe hallux valgus

Although the difference in HVA between the two groups of patients with mild and moderate hallux valgus was statistically significant, the numerical differences were not significant. Patients with severe hallux valgus did not have a significant difference in HVA between the two groups. In the congruency group, the TSP of patients with mild to moderate hallux valgus was lower than that of the incongruency group, while there was no significant difference between the two groups of patients with severe hallux valgus. In terms of DMAA, the mild patients had no significant difference between the congruency and incongruency groups, but for moderate-to-severe hallux valgus, the DMAA of the congruency group was greater than that of the incongruency group. Among all hallux valgus patients, the MPJA of congruency group was significantly lower than that of incongruency group, while the CI was significantly higher (Table 2).

**Table 2:** Comparison and analysis of different index of patients with different degrees of hallux valgus between congruency and incongruency groups

Groups	Index	Congruency	Incongruency	*T* value	*P* value
Mild	HVA (°)	23.60 ± 4.00	26.78 ± 2.88	− 4.172	0.000
	MTPJA (°)	7.41 ± 3.99	13.23 ± 6.58	− 3.744	0.001
	DMAA (°)	11.27 ± 5.69	14.20 ± 6.22	− 1.350	0.179
	CI	0.86 ± 0.06	0.78 ± 0.07	5.071	0.000
	TSP	3.55 ± 1.55	5.74 ± 0.81	− 9.265	0.000
Moderate	HVA (°)	32.98 ± 2.54	34.39 ± 2.86	− 2.329	0.022
	MTPJA (°)	7.57 ± 4.72	17.73 ± 6.05	− 8.309	0.000
	DMAA (°)	22.04 ± 7.77	16.22 ± 5.54	3.888	0.000
	CI	0.85 ± 0.06	0.75 ± 0.08	6.932	0.000
	TSP	4.37 ± 1.85	5.86 ± 1.09	− 4.320	0.000
Severe	HVA (°)	43.98 ± 1.89	47.30 ± 5.06	− 1.803	0.081
	MTPJA (°)	4.90 ± 3.32	29.12 ± 10.42	− 6.415	0.000
	DMAA (°)	32.45 ± 5.04	18.79 ± 10.00	5.154	0.000
	CI	0.80 ± 0.05	0.61 ± 0.15	5.925	0.000
	TSP	5.50 ± 1.41	6.27 ± 0.92	− 1.447	0.182

*HVA* hallux valgus angle, *DMAA* distal metatarsal articular angle, *MTPJA* the first metatarsophalangeal joint angle, *CI* congruency index, *TSP* tibial sesamoid position

**Fig. 3:**
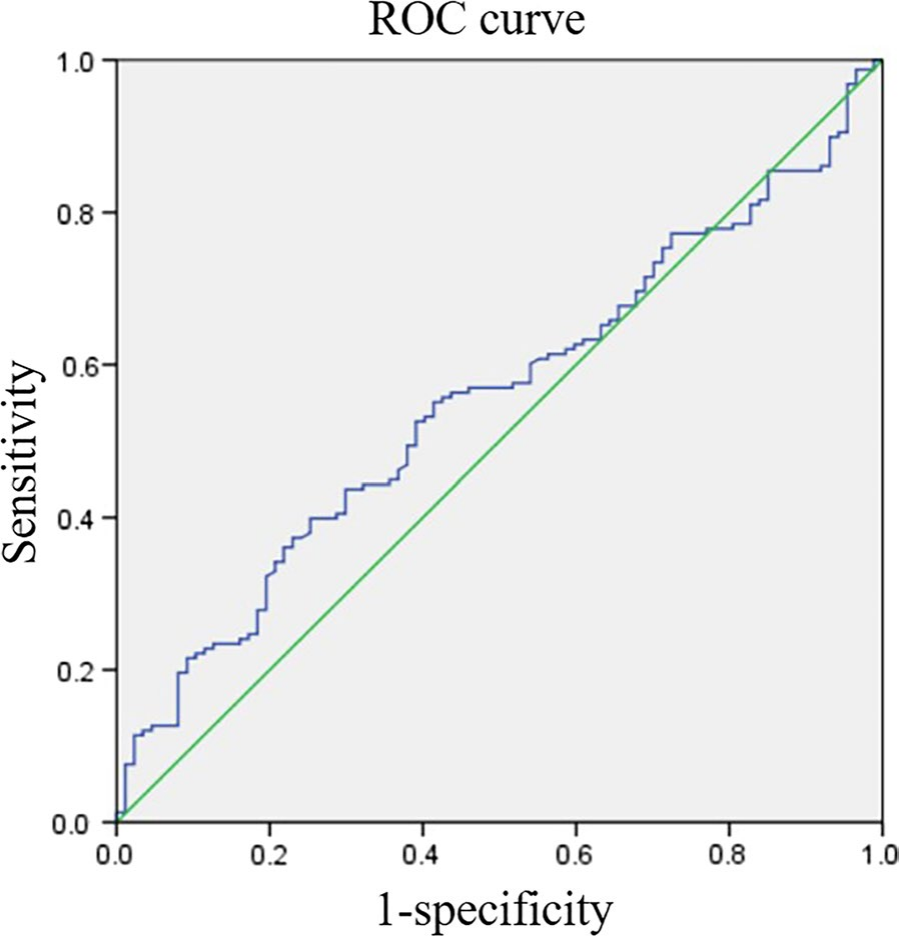
The ROC curve of DMAA and congruency of the first metatarsophalangeal (MTP) joint. The area under the curve (AUC) was 0.554

**Fig. 4:**
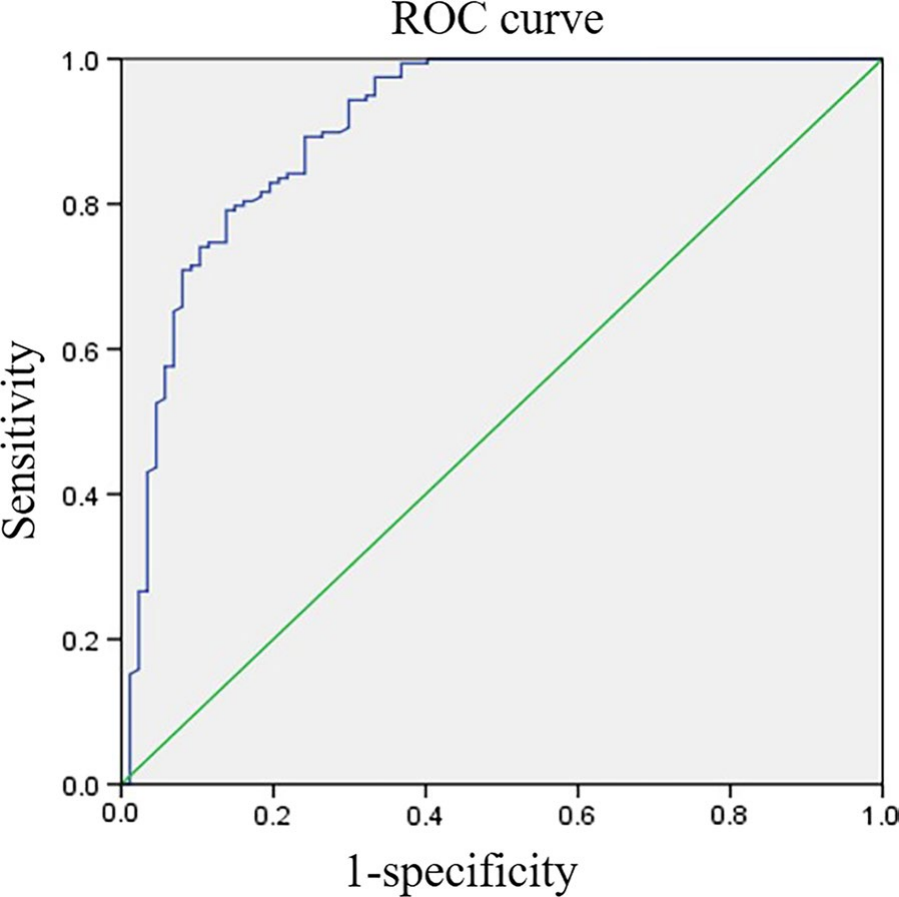
The ROC curve of the metatarsophalangeal joint angle (MTPJA) and congruency of the first metatarsophalangeal (MTP) joint. The area under the curve (AUC) was 0.906

**Fig. 5:**
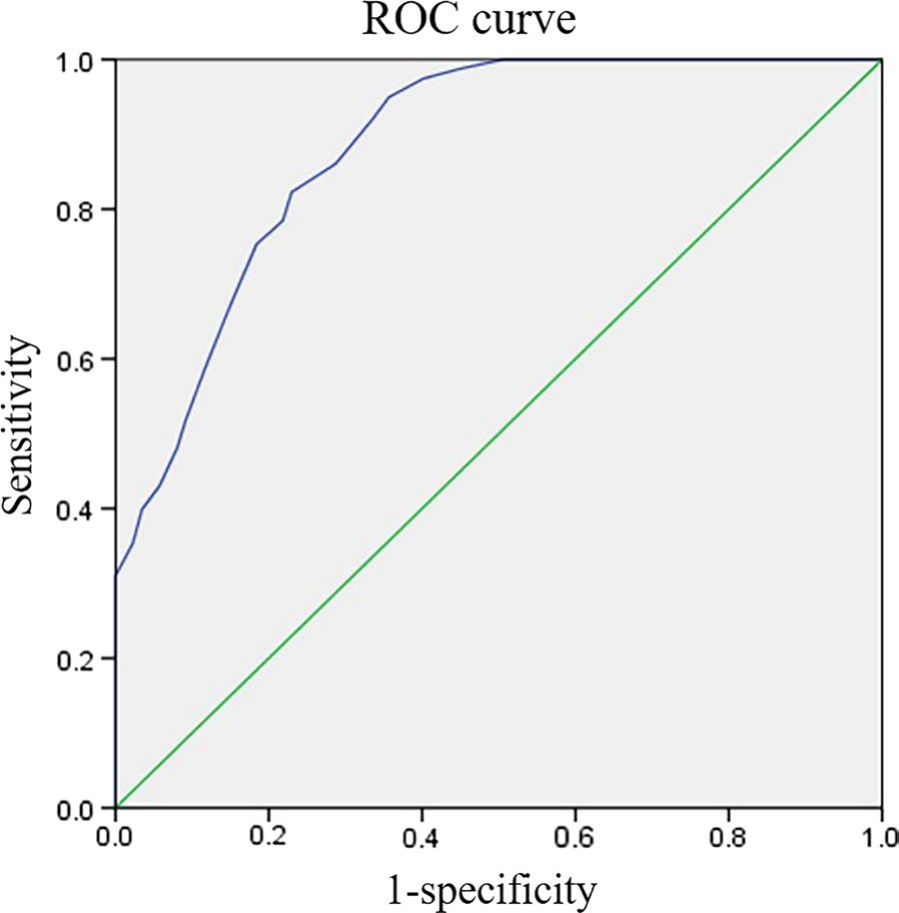
The ROC curve of the congruency index (CI) and congruency of the first metatarsophalangeal (MTP) joint. The area under the curve (AUC) was 0.884

Because of the significant difference in the DMAA, MTPJA and CI between the two groups of patients with moderate-to-severe hallux valgus (*P* < 0.001), we performed diagnostic tests and plotted the ROC curve with the DMAA, MTPJA and CI data. The AUC of the ROC curve for DMAA was 0.554 (*P* > 0.05) (Fig. 3). However, the MTPJA and CI were 0.906 and 0.884, respectively, which were both greater than 0.7 (*P* < 0.001) and showed significant diagnostic value (Figs. 4, 5). The sensitivity and specificity of the MTPJA reached 0.791 and 0.862, respectively, and the sensitivity and specificity of the CI reached 0.949 and 0.644. In addition, the critical value of the MTPJA was 10.67, and that of the CI was 0.765 (Table 3).

**Table 3:** Diagnostic test between two three index and congruency of the metatarsophalangeal joint

Index	Critical value	Sensitivity	Specificity	AUC	*P* value	Youden index	PPV	NPV	+ LR	− LR
DMAA	12.125	0.399	0.747	0.554	0.163	0.146	0.594	0.259	1.577	0.534
MTPJA	10.670	0.791	0.862	0.906	0.000	0.653	0.912	0.694	5.736	0.242
CI	0.765	0.949	0.644	0.884	0.000	0.593	0.829	0.875	2.664	0.079

*DMAA* distal metatarsal articular angle, *MTPJA* metatarsophalangeal joint angle, *CI* congruency index, *AUC* area under the curve, *PPV* positive predictive value, *NPV* negative predictive value, + *LR* positive likelihood ratio, − *LR* negative likelihood ratio

Correlation tests were performed on 5 parameters between the two groups of patients. In the congruency group, the DMAA was positively correlated with the HVA, while the correlation coefficients between the MTPJA, CI and HVA were low. In the incongruency group, the DMAA was not correlated with the HVA, while the MTPJA was positively correlated with the HVA, and the CI was negatively correlated with the HVA. The MTPJA and CI were negatively correlated in both the congruency group and the incongruency group (Table 4).

**Table 4:** Correlation test of different index between two groups

Index	MTPJA	DMAA	CI	TSP
*Congruency group*				
HVA	0.103 (0.197)	0.691 (0.000)	− 0.272 (0.001)	0.345 (0.000)
MTPJA	–	− 0.377 (0.000)	− 0.538 (0.000)	0.036 (0.654)
DMAA	–	–	0.057 (0.475)	0.517 (0.000)
CI	–	–	–	− 0.392 (0.000)
*Incongruency group*				
HVA	0.554 (0.000)	0.212 (0.048)	− 0.546 (0.000)	0.331 (0.002)
MTPJA	–	− 0.468 (0.000)	− 0.760 (0.000)	0.389 (0.000)
DMAA	–	–	0.322 (0.002)	− 0.101 (0.350)
CI	–	–	–	− 0.557 (0.000)

*HVA* hallux valgus angle, *DMAA* distal metatarsal articular angle, *MTPJA* the first metatarsophalangeal joint angle, *CI* congruency index, *TSP* tibial sesamoid position

## Discussion

The congruency of the first MTP joint plays an important role in the selection of the surgical method for post-operative recurrence in hallux valgus. Studies have shown that incongruency of the post-operative MTP joint was highly correlated with the recurrence of hallux valgus [11]. The previous literature reported [11] that the congruency of the first MTP joint has been evaluated only by a doctor’s visual assessment of whether the arcs of the MTP joint articular surface were parallel; however, there is no quantitative indicator. In addition, the DMAA is often used to assess dislocation of the first MTP joint [14]. For example, for patients with severe hallux valgus accompanied by an increased DMAA, double metatarsal osteotomy (DMO) is an effective surgical method [15]. However, due to the related complications, such as shortening the length of the first metatarsal bone, post-operative avascular necrosis of the metatarsal head and metastatic metatarsalgia, the application of this technique has been limited to a certain extent [16]. Wang's study compared the efficacy of rotating scarf osteotomy and DMO for hallux valgus accompanied by an increased DMAA. It was believed that there was no significant difference between the two methods, but the former had a lower incidence of complications [16]. Jeong [17] used the point-connecting method to measure the HVA and intermetatarsal angle (IMA) because with the traditional central axis measurement method, it was difficult to reflect the congruency of the MTP joints.

Evidence suggests that the DMAA is not suitable for use in evaluating the congruency of the first MTP joint [18]. For the larger metatarsals of the DMAA, there will also be two situations where the MTP joints are congruent and incongruent. Our statistical results showed that there was an obvious relationship between the patients’ DMAA and the congruency of the first MTP joint for moderate-to-severe hallux valgus. The DMAA of the congruency group was significantly larger than that of the incongruency group. The ROC curve results showed that the AUC was only 0.554 and that the P value was 0.163, indicating that the use of DMAA cannot accurately assess congruency of the first MTP joint. In terms of the correlation test, the DMAA and HVA were positively correlated in the congruency group, while there was no correlation between the two in the incongruency group. This indicates that if the DMAA is to be used to assess the degree of hallux valgus, it is limited to the congruency group. In patients with significant dislocation of the first MTP joint, the DMAA is less effective in assessing the severity of hallux valgus.

We innovatively proposed two quantitative evaluation indexes for congruency of the first MTP joint on weightbearing foot anterior–posterior images, the MTPJA and CI, which were quantitatively assessed by measuring the angles of the articular surfaces at both ends and the degree of bonding of the articular surfaces (Fig. 1). Because our data were not clinically necessary, many asymptomatic patients were also included, resulting in a larger proportion of patients with mild hallux valgus, most of whom had congruent MTP joints. The proportions of patients with congruency and incongruency with moderate hallux valgus were basically the same. For patients with severe hallux valgus, nearly a quarter of them had congruency of the first MTP joint, which is similar to the data reported by Coughlin [19]. In addition, as the severity of hallux valgus gradually increased, the MTPJA gradually increased, and the CI gradually decreased, indicating that the contact surface of the first MTP joint surface will gradually decrease.

In comparing the difference between the congruency and incongruency groups, there was no difference in the HVA between the two groups of patients with mild, moderate or severe hallux valgus. However, among patients with moderate-to-severe hallux valgus, the difference between the MTPJA and CI was large; moreover, the CI of the congruency group was greater than that of the incongruency group, and the MTPJA of the congruency group was smaller than that of the incongruency group, indicating that the MTPJA and CI could be used to effectively assess the congruency of the first MTP joint. ROC curves of the MTPJA and CI showed that the AUCs were 0.906 and 0.884, respectively, and that both had diagnostic power. The critical value of the MTPJA was 10.67, and that of the CI was 0.765. We can thus define the first MTP joint as incongruent if the value of MTPJA is greater than 10° or CI is less than 0.765, and the degree of incongruency can be measured by the specific value of the two. That is, the larger the MTPJA and the smaller the CI, the greater the degree of congruency is. In the congruency group, the correlation coefficients between the MTPJA, CI and HVA were low, while in the incongruency group, the MTPJA and HVA were significantly positively correlated and the CI and HVA were negatively correlated; that is, the more severe the hallux valgus is, the more deviated the normal ranges of the MTPJA and CI are. Of course, the smaller the value of the MTPJA and the larger the value of the CI, the better the matching relationship of hallux valgus is. Therefore, whether in the congruency group or incongruency group, there was a significant negative correlation between the two. In our previous article [6], 36 patients (38 feet) with moderate-to-severe hallux valgus were followed up at different times before and after surgery, and their CI recovered from 0.75 before surgery to 0.95 at the last follow-up. Similar to the HVA, IMA, etc., all of the indicators recovered well, which also verifies the effectiveness of this indicator to a certain extent.

The present paper also has some limitations that should be taken into consideration. First, this study focused only on the statistical analysis of radiological parameters and did not apply the MTPJA and CI to the comparison of parameters before or after the operation in hallux valgus patients. It also did not classify the magnitude of the two parameters relative to the clinical symptoms. This is what we need to include in the next step of our research. In addition, the patients included in this study had a certain deviation. The number of patients with mild hallux valgus was too large, but because metatarsophalangeal joint mismatch mostly occurs in patients with moderate-to-severe hallux valgus, we believe that the data in the study are still reliable.

In summary, in hallux valgus of different degrees, especially in patients with moderate-to-severe hallux valgus, the first MTP joint is either congruent or incongruent. The DMAA has poor performance in evaluating matching relationships, and the previously used imaging indicators are only qualitative evaluations. The MTPJA and CI can be used to quantitatively evaluate the congruency of the first MTP joint, and 10° and 0.765 are used as the demarcation points. Clinically, it is necessary to consider the congruency of the first MTP joint in the selection of different degrees of hallux valgus surgery. The MTPJA and CI can be used as quantitative evaluation indicators.

## Data Availability

The datasets used during the current study are available from the corresponding author on reasonable request.
